# Magnetic, electrical and mechanical properties of Fe_40_Mn_40_Co_10_Cr_10_ high entropy alloy

**DOI:** 10.1038/s41598-021-87527-x

**Published:** 2021-04-13

**Authors:** M. Egilmez, W. Abuzaid

**Affiliations:** 1 Department of Physics, American University of Sharjah, Sharjah, UAE; 2 Department of Mechanical Engineering, American University of Sharjah, Sharjah, UAE

**Keywords:** Magnetic properties and materials, Mechanical engineering

## Abstract

A prototypical, single-phase, and non-equiatomic high entropy alloy Fe_40_Mn_40_Co_10_Cr_10_ has been mechanically deformed at room and cryogenic temperatures. Plastic deformation was accommodated via crystallographic slip at room temperature while transformation induced plasticity (TRIP) has been observed in samples deformed at 77 K. The stress-induced martensitic transformation occurred from face-centered cubic (FCC) to hexagonal close-packed (HCP) structures. A detailed electron backscatter diffraction analysis was utilized to detect phase change and evaluate the evolution of the HCP phase volume fraction as a function of plastic strain. Physical properties of undeformed and deformed samples were measured to elucidate the effect of deformation-induced phase transitions on the magnetic and electrical properties of Fe_40_Mn_40_Co_10_Cr_10_ alloy. Relatively small magnetic moments along with non-saturating magnetic field dependencies suggest that the ground state in the considered material is ferrimagnetic ordering with coexisting antiferromagnetic phase. The temperature evolution of the coercive fields has been revealed for all samples. The magnitudes of the coercive fields place the considered system into the semi-hard magnetic alloys category. The temperature dependence of the zero-field cooled (ZFC) and field cooled (FC) magnetization was measured for all samples in the low field regime and the origin of irreversibility in ZFC/FC curves was discussed. Besides, the temperature dependence of the resistivity in all samples was measured and the possible conduction mechanisms were discussed.

## Introduction

High entropy alloys (HEA) have received a great deal of attention over the past decade because of their potential to provide exceptional mechanical and physical functionalities^1–5^. In these alloys, initially, it was suggested that the presence of multiple elements (often 5 or more) with near equiatomic ratios would maximize the configurational entropy to a level sufficient to overcome the compound formation enthalpy and subsequently stabilize the solid solution state with a uniform face-centered cubic (FCC) or body-centered cubic (BCC) crystal structures^1^. However, recently the concept of the maximization of the configurational entropy through the mixing of multiple elements with near equiatomic ratios has been questioned as new experiments and *ab*-initio based theoretical studies revealed that the configurational entropy maximization is not the most essential parameter when designing multicomponent alloys^1,2^. It was suggested that the high entropy criterion could be supplemented with a low enthalpy criterion. Practically, the low enthalpy criterion was very attractive as it offers a wider compositional range that comprises alloys with fewer elements as well as alloy structures that deviate from equiatomic compositions^6–9^. Fe_80-x_Mn_x_Co_10_Cr_10_ (at%) is a prototype quaternary system of non-equiatomic alloys with tailored mechanical properties^10^. In this alloy, the Co and Cr contents are deliberately kept significantly lower than the equiatomic fractions to avoid the formation of a Cr-rich σ—intermetallic phase. In this quaternary structure, the variation in the manganese (Mn) content leads to several deformation mechanisms including dislocation dominated plasticity, twinning induced plasticity (TWIP), and transformation induced plasticity (TRIP)^10^. In particular, in Fe_80-x_Mn_x_Co_10_Cr_10_ (at %) when Mn content was at 45% and 40%, a single-phase face-centered cubic (FCC) structure was stabilized while Mn content less than 35% leads to the formation of hexagonal closed packed (HCP) structures within the FCC matrix.

Among all the compositions studied, Fe_40_Mn_40_Co_10_Cr_10_ is particularly interesting as a homogenous solid solution with intriguing mechanical properties forms^6,10^. At a relatively high deformation temperature (i.e., room temperature), plasticity is accommodated in this single phase FCC system through planar dislocation slip at true strain levels of less than 10% whereas deformation twinning is induced at higher strains exceeding 10%. The activation of deformation twinning enhances the hardening rates and capacity similar to TWIP steels such as FeMnAlC and FeMnC. At cryogenic temperatures, deformation is accommodated primarily through FCC–HCP martensitic transformation (TRIP) resulting in higher strength and ductility magnitudes compared to room-temperature deformation. The attained strength and ductility levels, as well the operative deformation mechanisms are heavily influenced by the microstructure, especially grain size and orientation^6^.

However, and despite extensive work on the metallurgical, mechanical, and thermodynamic properties of the nonequiatomic FeMnCoCr system, there is limited work dedicated to investigating the physical properties^11,12^. Most of the studies on magnetic and electrical properties of high entropy alloys focus on equiatomic FeCoCrNi*X* alloy (i.e., derivatives of the FeCoCrNiMn Cantor alloy) where *X* is Mn, Ti, Cu, or Pd and non-equiatomic FeCoCrNi with slight doping of Al, Cu, and Si^13–19^. A wide spectrum of magnetic properties has been reported ranging from soft to semi-soft magnetic behavior in their coercivity^3^. Among these alloys, the most studied single-phase FCC Cantor alloy exhibited multiple magnetic transitions from disordered paramagnetic phase to spin glass and subsequently to an ordered ferromagnetic state at low temperatures^15^. More interestingly, the anti-invar property of this alloy which in turn induces strong magnetism in this alloy has been recently reported^20^ pointing to a potential for magnetism related applications. The efforts in this work have been focused on the magnetic and transport properties of Fe_40_Mn_40_Co_10_Cr_10_ high entropy alloy. This prototype high entropy alloy exhibits intriguing mechanical properties with simultaneous enhancements in strength and ductility at cryogenic temperatures. Such enhancements in properties have been attributed to the TRIP effect at low deformation temperatures. It remains unclear, however, how this change in deformation mechanism (i.e., slip dominated at high temperatures and martensitic transformation at low deformation temperatures) would impact the physical properties of deformed Fe_40_Mn_40_Co_10_Cr_10_. This aspect will be a subject of investigation in the current study.

## Results and discussion

**Figure 1:**
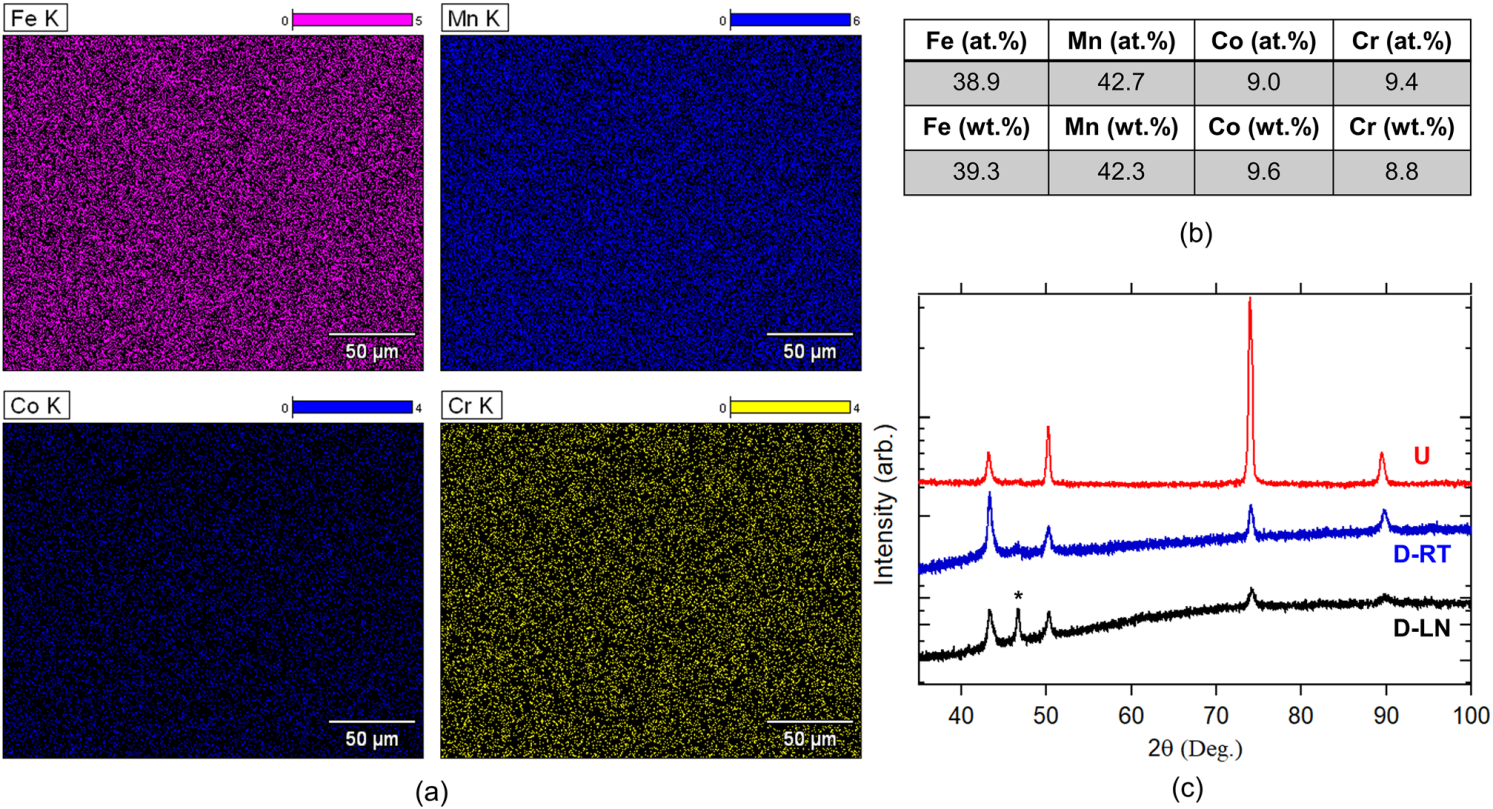
(**a**) Electron dispersive X-ray elemental composition area maps for constituent elements. (**b**) Atomic and weight distributions of the constituent elements. (**c**) X-ray diffraction data for pristine (U) and deformed samples. HCP peaks are marked with*.

Electron dispersive X-ray (EDX) analysis has been carried out to determine elemental distribution at the surface of the specimen. The area scans presented in Fig. 1a revealed a uniform distribution of elements and a homogeneous chemical composition over the entire specimen. Data collected at higher magnifications (double and triple the magnification compared to Fig. 1a) also confirms the homogeneity of the composition. Nominal atomic and weight compositions of the actual specimen are given in Fig. 1b. X-ray diffraction measurements obtained with CuK-α1 radiation are shown in Fig. 1c. For the undeformed condition, the visible peaks indicate a single FCC phase with no parasitic phases. The corresponding lattice parameter was 3.41(1) Å. The obtained XRD spectrum is in line with previously reported results on the same composition^10^. The XRD data includes the spectrum of the deformed samples which will be discussed later.

**Figure 2:**
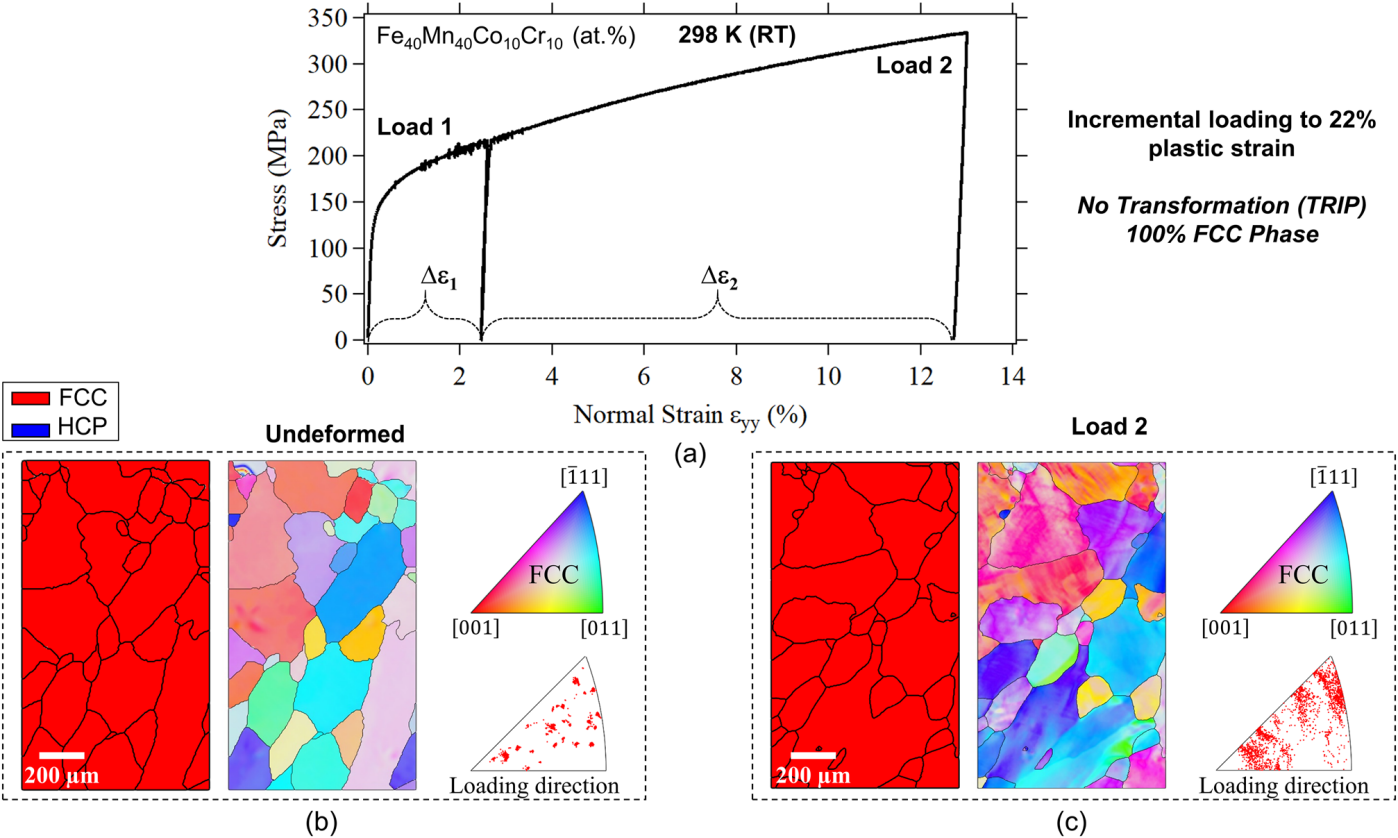
(**a**) Stress–strain curves following incremental loading at room-temperature (RT). (**b**) Phase map and grain orientation map obtained from the gauge section of the specimen before deformation. (**c**) Phase and grain orientation maps collected from the same area shown in (**b**) following the shown loading history (about 13% strain). Undeformed and deformed conditions point to FCC single phase with no signs of phase transformation.

One of the objectives of the current work is to further elucidate the correlation between mechanical deformation and physical properties of the considered FeMnCoCr HEA. In particular, in the case of the desirable TRIP effect at low deformation temperatures. Figure 2 shows the incremental stress–strain curve for a polycrystalline specimen deformed at room temperature. The phase and grain orientation maps were captured using electron backscatter diffraction (EBSD) before loading (shown in Fig. 2b) as well as after about 13% (shown in Fig. 2c) and 22% plastic strains. As expected, the deformation was accommodated primarily via crystallographic slip with no signs of phase transformation at this loading temperature.

**Figure 3:**
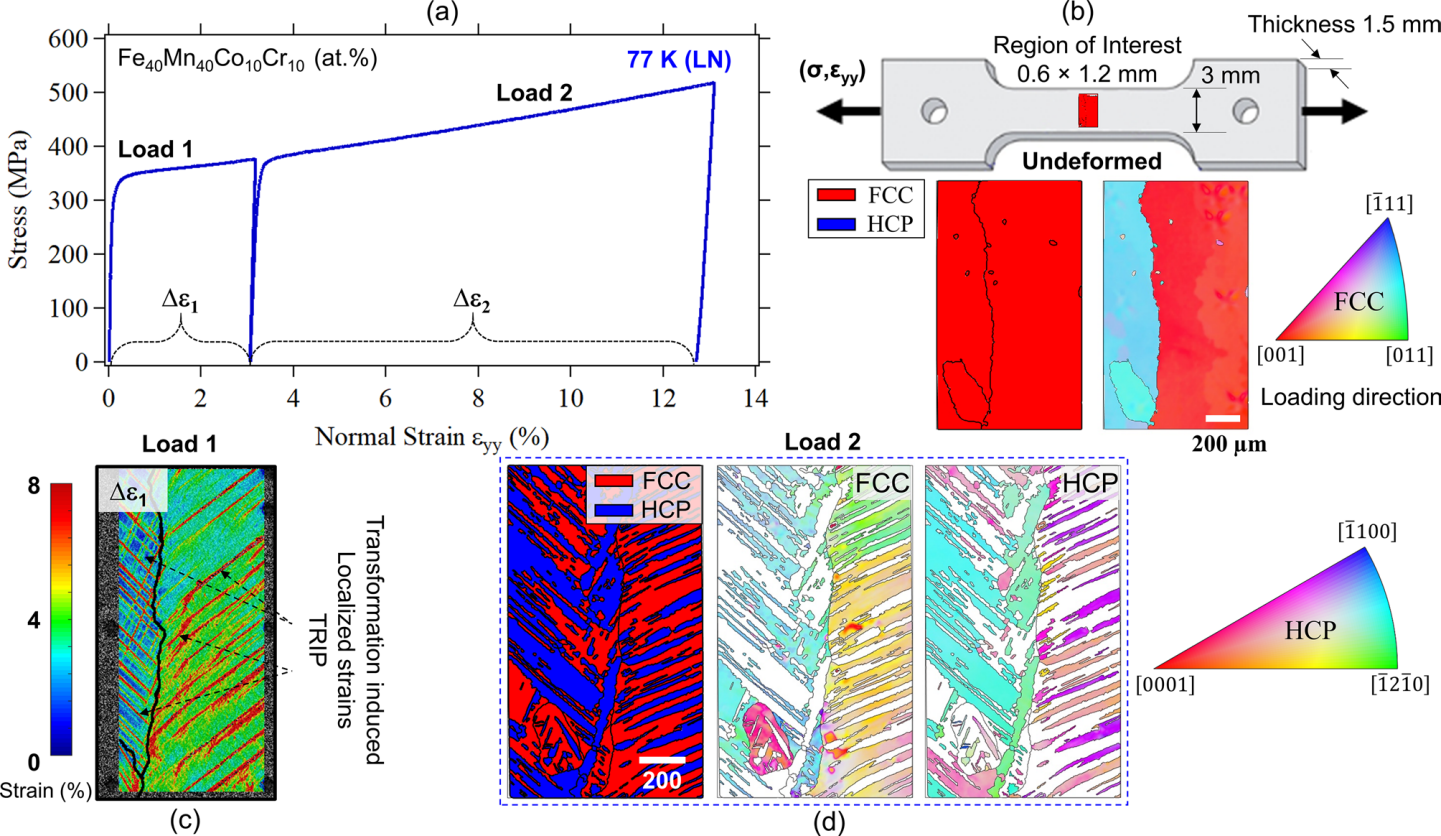
(**a**) Stress–strain curves following incremental loading at 77 K (submerged in liquid nitrogen LN). (**b**) Phase map and grain orientation map obtained from the gauge section of the specimen prior to deformation. (**c**) High resolution strain contour plots showing the residual strain field in the region of interest following the first deformation increment (Load 1). The localized strains exhibit very high levels and are associated with transformation induced plasticity (TRIP). (**d**) Phase and grain orientation maps collected from the same area shown in (**b**) following the shown loading history (about 13% strain). The presence of both FCC (original phase) and HCP (TRIP) phases is clear from the EBSD phase maps at this deformation temperature.

**Figure 4:**
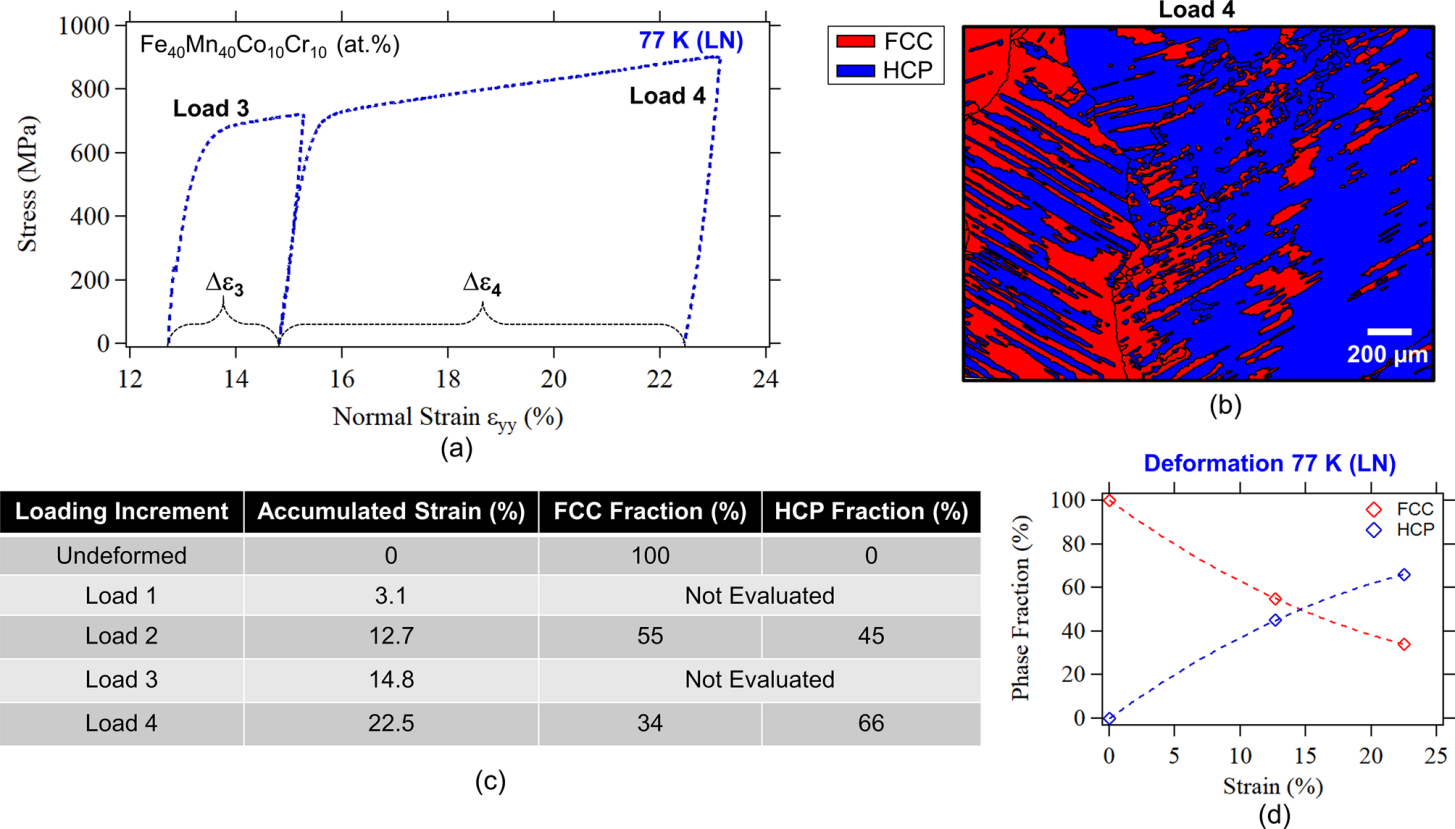
(**a**) Stress–strain curves following incremental loading at 77 K. Deformation increments Load 3 (13–15% strain) and Load 4 (15–22.5% strain) are shown. Previous loading cycles are presented in Fig. 3. (**b**) Phase map obtained from the gauge section following Load 4 which corresponds to a total of 22.5% plastic strain. The FCC and HCP phase volume fractions as a function of loading are summarized in Table (**c**) and plot (**d**).

The stress–strain response obtained at the lower deformation temperature (i.e., 77 K) is shown in Fig. 3. EBSD data was collected from the sample’s gauge Sect. (0.6 × 1.2 mm^2^) prior to loading as shown in Fig. 3b. The phase map indicates a single phase with an FCC structure. Compared to the RT sample, the strength levels at 77 K were significantly higher. At comparable strain levels (i.e., about 12% plastic strain), the phase and grain orientation maps point to significant accumulation of HCP phase post loading (see FCC and HCP grain orientation maps in Fig. 3d). High-resolution strain measurements were also collected following the procedure detailed in^21,22^. Figure 3c shows a high-resolution strain contour plot obtained following the first loading increment (Load 1). The clear strain bands (high strain regions) highlight the significant strain localization associated with phase transformation and TRIP effect (note the similar slopes for the localized strain bands in Fig. 3c and the martensite variants phase boundaries in Fig. 3d). The HCP phase fraction increased with additional loading as shown in Fig. 4. Throughout the multiple loading increments, the fraction of the HCP phase increased from 0, before loading, to 66% following 22.5% plastic strain. Note that the presence of the HCP phase was further confirmed by the XRD data (in the spectra, peaks belonging to the HCP phase are marked with * (see Fig. 1c). However, it should be pointed out that the final volume fraction of the HCP phase has been shown to exhibit grain size dependence for this alloy system^23^.

As detailed above, TRIP effect was observed for the Fe_40_Mn_40_Co_10_Cr_10_ alloy at cryogenic deformation temperatures. Mechanical deformation at 77 K led to a significant structural transformation from a crystal structure of FCC to HCP. Detailed analysis based on EBSD revealed critical information on the volume fraction of HCP phase as a function of strain. Such structural transformation is further evidenced and visible in the XRD spectrum shown in Fig. 1c as HCP peaks started to appear in the deformed samples (LN). Peaks that belong to the HCP phase were marked with (*) in the spectrum. One important observation here is that the measurements collected post deformation exhibited substantial peak intensity suppression as well as full width half maximum broadening. Such observation can be taken as a clear indication of the presence of a larger amount of defects like dislocations and stacking faults. This was observed for both of the considered deformation temperatures, RT and LN.

**Figure 5:**
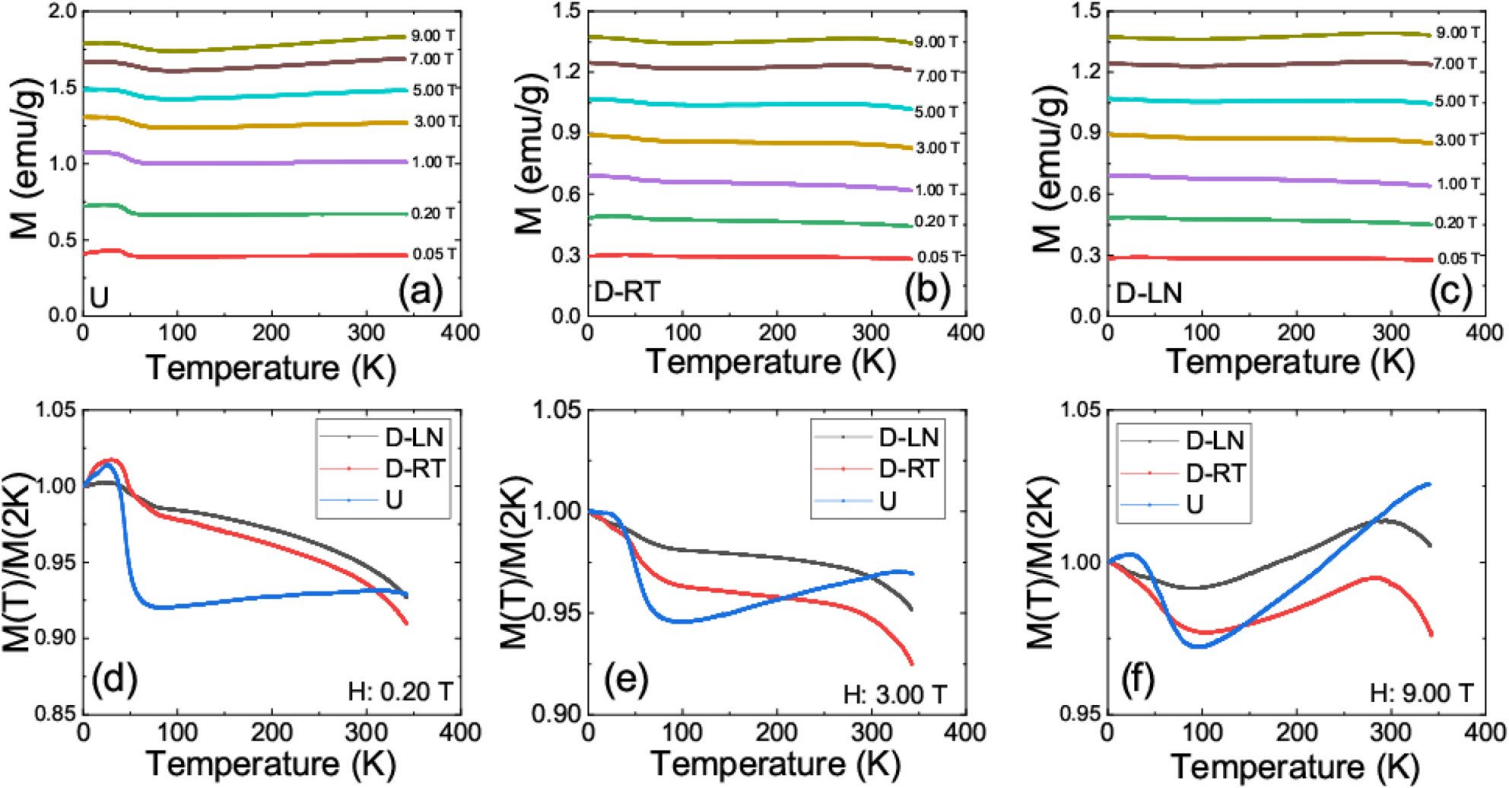
Temperature dependence of the zero field cooled magnetization in indicated fields for (**a**) undeformed FeMnCoCr, (**b**) Room temperature deformed FeMnCoCr, (**c**) Liquid Nitrogen deformed FeMnCoCr. Temperature dependence of the zero field cooled magnetization normalized to its value at 2 K for undeformed and deformed samples in (**c**) 0.20 T (**d**) 3.00 T (**e**) 9.00 T.

The reported mechanical properties are of great interest for this relatively new group of materials which are being considered for structural and load-bearing applications at cryogenic temperatures. Besides, the presence of magnetic elements places these materials in a group of materials with possible magneto-transport functionalities. Magnetic and transport properties of undeformed and deformed Fe_40_Mn_40_Co_10_Cr_10_ would be of great interest since the magnetic properties for the FCC and HCP dominant phases of the same composition can be revealed. Magnetic properties of various high entropy alloys with different crystallographic structures have been the subject of some theoretical works but to the best of the authors knowledge, experimental studies are limited^18,24–26^. As mentioned above, the discussion in this study will focus on undeformed (U), room temperature deformed (D-RT) and Liquid nitrogen deformed (D-LN) samples. Both of the deformed specimens (D-RT and D-LN) were investigated at similar levels of plastic strain. Temperature dependencies (2–350 K) of the zero-field cooled (ZFC) magnetizations of the undeformed sample measured at different magnetic fields are shown in Fig. 5a.

The measurements presented in Fig. 5 revealed that the magnetic ordering temperature (T_c_) of the material is much higher than the measurement limits of the utilized experimental setup (i.e., T_c_ exceeding 350 K). The measured magnetization for the undeformed sample at 2 K was around 1.8 emu/g in a field of 9 T and dropping to only 0.4 emu/g in a field of 0.05 T. Magnetizations ranging between 0.2 emu/g and 80 emu/g have been previously reported for high entropy alloys^3,24,27,28^. Hence, the observed magnetizations are consistent with existing literature. It should be pointed out that the overall magnetization of any multi magnetic element compound, in general, will be determined by the complex magnetic interactions of constituent elements. The quaternary system considered in this work consist of 40 at.% Fe and 40 at.% Mn and there exists a vast amount of literature on Fe/Mn compounds in which the magnetic moments of the manganese tend to align antiparallel to the magnetic moment of the Fe which in return reduces the overall magnetic moment of the material^29–31^. Moreover, Cr magnetic moments tend to align antiparallel with Fe and Co^32^. Note that the observed bulk magnetizations correspond to 0.0177 μ_B_/f.u. magnetic moment at 2 K and 9 T, here the formula unit (f.u.) is defined as Fe_0.4_Mn_0.4_Co_0.1_Cr_0.1_. This rather small magnetic moment seems to be much lower than the individual magnetic moments of constituent atoms. A strong magnetic frustration due to the fluctuations of Mn and Cr magnetic moments and their antiferromagnetic coupling to Fe and Co is expected to significantly reduce the overall magnetic moment of the system since these atoms correspond to 50% of the structure. Recent X-ray magnetic circular dichroism (XMCD) studies on high entropy alloys showed that that the bulk magnetic moments can be strongly suppressed with increasing concentration of antiferromagnetically coupled elements in the solid solution^33^.

In the temperature dependence of the magnetization shown in Fig. 5, there is a clear up turn around 70 K and reaching saturation at around 40 K. Interestingly, this behavior is insensitive to the increasing magnetic fields. Temperature dependence of the magnetization is almost flat between 70 and 290 K with a slight downward turn in magnetization around 290 K. Note that this region significantly changes with increasing field. The temperature dependence of the ZFC magnetizations were also measured for deformed samples as shown in Fig.5b,c. The magnetic moment dropped significantly for the deformed specimens, both RT and LN deformed in comparison to the undeformed specimen. In an attempt to clarify the relative changes in magnetization, the temperature dependence of the magnetizations were normalized by the corresponding value at 2 K as shown in Fig. 5d–f. The transition at 70 K, which was clear in the undeformed specimen, was smeared out for D-LN and D-RT samples. This change was substantial at low fields but became insignificant at relatively large magnetic fields. Schneeweis et al. observed an abrupt change in the magnetic moment for CrMnFeCoNi at around 40 K, similar to ones reported here (in this work onsetting around 70 K), and assigned it to the ferromagnetic transition in the Cantor alloy. In addition, their magnetization data at higher temperatures suggested that a spin glass phase transition was happening at around 93 K^15^. In the considered samples, very small magnetic moments suggest that the antiferromagnetic couplings between Mn, Cr, Fe, and Co are very strong in suppressing the bulk magnetization. However, that antiferromagnetic coupling is not strong enough to diminish the magnetic state completely, within the limits of the measurement range. Hence, the data suggests that the material is in a weakly ordered magnetic state, possibly in a ferrimagnetic ground state, at all accessible temperatures. Note that a ferrimagnetic ordering is a type of magnetic ordering in which populations of atoms with opposing magnetic moments (as in antiferromagnetism) are unequal in magnitude therefore a spontaneous small magnetization remains in the material. The observed transition at 70 K marks the onset where the antiferromagnetic coupling weakens (ferromagnetic coupling increases) and about a 10% increase in the bulk magnetization is observed in the undeformed sample (see Fig. 5d). Note that such an increase in the magnetization at 70 K is about 3.5% for D-RT (FCC phase) and drops to only 1.5% for D-LN specimen (FCC and HCP phases). Based on this scenario, the antiferromagnetic couplings in the HCP containing samples are the strongest. This observation is in line with recent DFT calculations where an increased antiferromagnetic coupling is anticipated when a sample transforms from FCC to HCP phase^18^. However, one has to mention here the role of the increased defect structure associated with mechanical deformation. The 70 K abrupt change is significantly smeared out also in the D-RT sample indicating that the defect state of the sample is likewise very important in defining the magnetic ground state of this group of materials. On the other hand, the magnetic field response of magnetization above and below 70 K is significantly different for all samples. As revealed in Fig. 5e,f at higher fields, the high temperature ferromagnetic phase grows much faster with increasing magnetic field than the low temperature one resulting in the observed behavior in the 9 T ZFC measurements. As mentioned above, deformed specimens have a significantly lower magnetic moment than the undeformed sample.

The D-LN sample consists of more than 50% HCP phase, hence a significant drop in volume magnetization can be understood. Niu et al*.* performed detailed density functional theory calculations on ternary equiatomic CrCoNi and quinary CrMnFeCoNi for the FCC and the HCP crystal structures. In the ternary alloy, it was reported that the Ni and Co acquire positive magnetic moments^18^. Cr magnetic moments are typically negative in the HCP phase, however, shift to positive values in certain FCC configurations^18^. On the other hand, in the quinary alloy, the magnetic moments of Ni and Co show similar behavior as in the ternary alloy, while Cr moments show a wider range of fluctuations. More relevant to the FeMnCoCr system considered here, Fe and Mn atoms have more complicated trends; Mn moment switches between positive and negative values in both phases, and Fe appears to experience negative magnetic moments in the HCP structure. As mentioned before, the magnetic relationship between Fe and Mn atoms has been the subject of numerous studies which point that composition and defect structure can have an impact on the magnetic interaction^30^. In particular, a recent study revealed that the presence of a vacancy near the Mn atom, inducing a local charge decrease, tends to favour the antiferromagnetic Fe–Mn interaction while the presence of an interstitial impurity with a strong electronic hybridization with Mn can favour a ferromagnetic Fe–Mn interaction^31^. Consistent with this, Ma et al*.* reported that Mn magnetic moments are antiferromagnetically oriented with respect to Fe and Co moments for unit cell volumes slightly higher than the equilibrium volume^24^. On the other hand, Schneeweiss et al*.* reported that when pure Mn, as an antiferromagnetic element, combines with Fe, Co, and Cr, the preferred antiferromagnetic state of the Mn atoms cannot be completely fulfilled as Mn in the FCC structure would be magnetically frustrated due to geometric constraints on nearest neighbour exchange interactions^15^. This statement is further supported by their density functional theory simulations: after full relaxation of structural and magnetic parameters, only five Mn atoms flipped their magnetic moments into the antiferromagnetic orientation while the remaining 13 Mn atoms kept their original ferromagnetic orientation^15^. In addition to the complex magnetic relation between Fe and Mn, the magnetic interactions that arise from alloying Fe and Cr have been the subject of many experimental and theoretical studies. It has been reported that a number of magnetic states can be formed based on the Cr concentration in the FeCr system ranging from magnetic frustration to antiferromagnetic order^32,34–38^. Klaver et al*.* reported that at dilute concentration levels of Cr in the Fe matrix have their magnetic moments aligned antiferromagnetically with ferromagnetic Fe^35^. However, when the concentration of the Cr is larger than the dilute limit but still low (similar to the FeMnCoCr system considered in this work) magnetic frustration takes place as evidenced from the sign change in the magnetic moment of the Cr atoms for various nearest neighbor interactions. Considering the fact that the considered alloy consists of 40% Fe, 40% Mn, 10% Co and 10% Cr, the Mn and Cr interaction may also play an important role in overall magnetic properties. In Mn/Cr alloys, it is conveyed that Mn prefers antiferromagnetic alignment while Cr is responsible for short ranged ferromagnetism. One can iterate the discussion based on the Fe with Mn and Cr to Co case with similar conclusions. It is obvious that the magnetic interaction in the investigated specimens is very complicated: there exist many competing interactions between ferromagnetic Fe, Co, and anti-ferromagnetic Mn and Cr. Moreover, the local defects play a significant role in the determination of the volume magnetization. Consistent with this, Wu et al. recently reported that stacking fault energy and magnetism is interlinked in a nonequiatomic quinary Mn-consisting high entropy alloy^12^.

**Figure 6:**
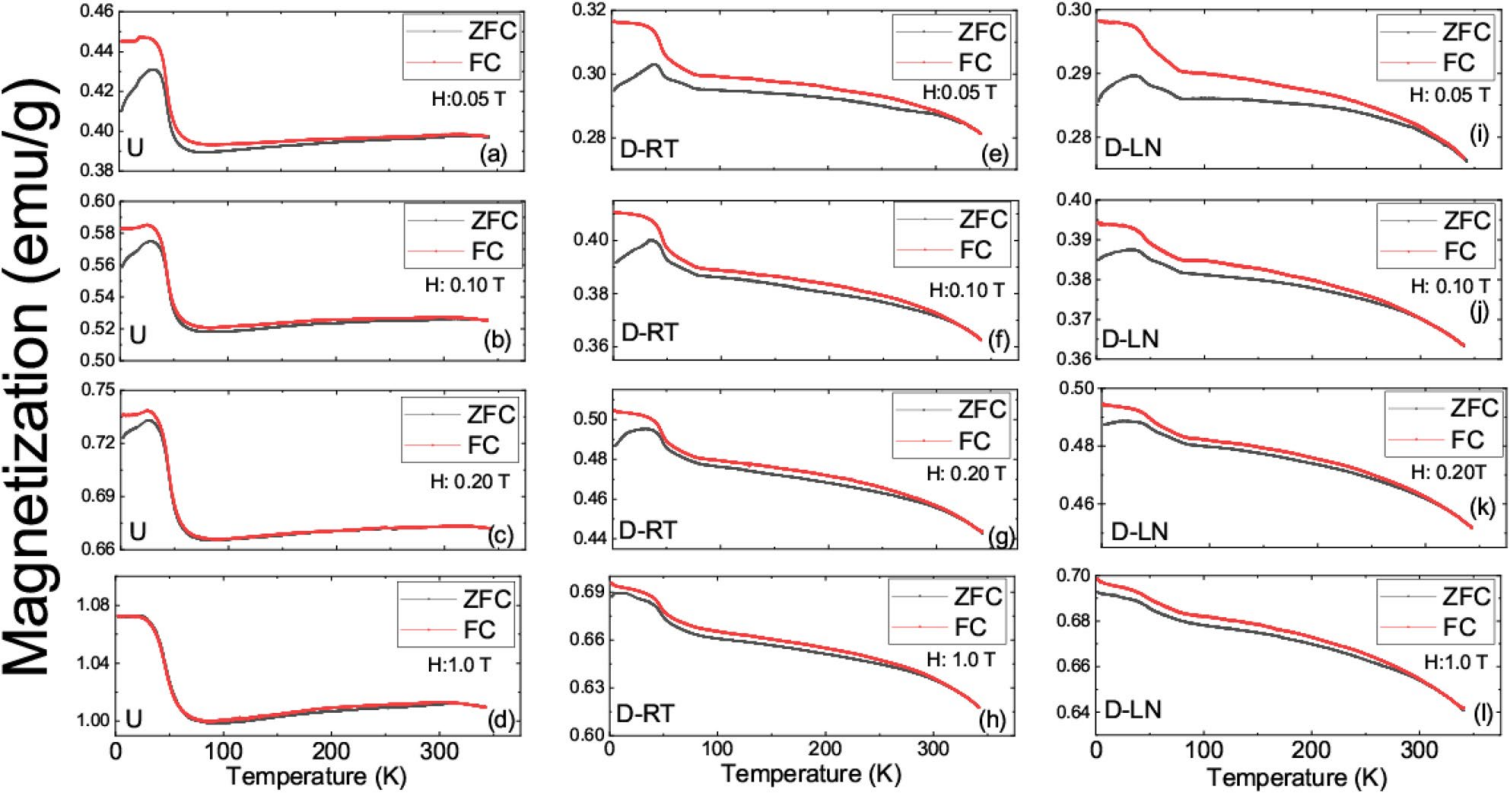
(**a**–**l**) Temperature dependence of the zero field cooled (ZFC) and field cooled magnetizations (FC) at indicated fields for undeformed and deformed samples.

The temperature dependence of the ZFC magnetizations contrasted with field cooled (FC) magnetizations are shown in Fig. 6 for all the samples in the low magnetic field regime. Before each set of measurements, each sample was cooled in zero magnetic field to 2 K and ZFC data was collected in the warming up cycle while the FC data has been collected in the cooling down cycle. In magnetic fields less than 0.20 T, irreversibility in magnetization is visible for all samples with a visible peak in ZFC measurements. This peak occurs at 32 K for the undeformed sample and at 38 K and 37 K for the D-RT and D-LN specimens, respectively. The difference between ZFC and FC curves are often attributed to magnetic clusters, spin glass state, or anisotropy in the sample. The occurrence of a possible spin glass state or magnetic frustration due to the presence of Mn and Cr has been discussed for the CrMnFeCoNi system by Wu et al*.* and revealed the relationship between the stacking fault energy and magnetic ordering^12^. More relevant to this study, Wu et al*.* evidenced the role Mn on the stabilization of antiferromagnetic ordering in their system. In addition to the above, studies reported by Schneeweiss et al*.* show clear evidence of spin-glass state^15,39^. Similar to their work, the results reported in this study at low temperatures indicated that the specimens exhibit magnetic relaxation effects suggesting that magnetic frustration is creating a non-equilibrium state. Interestingly, the irreversibility in ZFC and FC curves becomes insignificant in the undeformed samples at high magnetic fields which possibly suggests that larger fields become sufficient to overcome the energy barrier between nonequilibrium configurations of the magnetic moments. Unlike the undeformed sample, at higher fields, the irreversibility in ZFC and FC curves persists in deformed samples (see Fig. 6). It is a known fact that deformation will result in elongation in the loading direction and will result in anisotropy in physical and mechanical properties. Hence, the increase in the irreversibility of the ZFC/FC magnetizations in deformed samples is possibly related to the deformation induced anisotropy in the samples.

**Figure 7:**
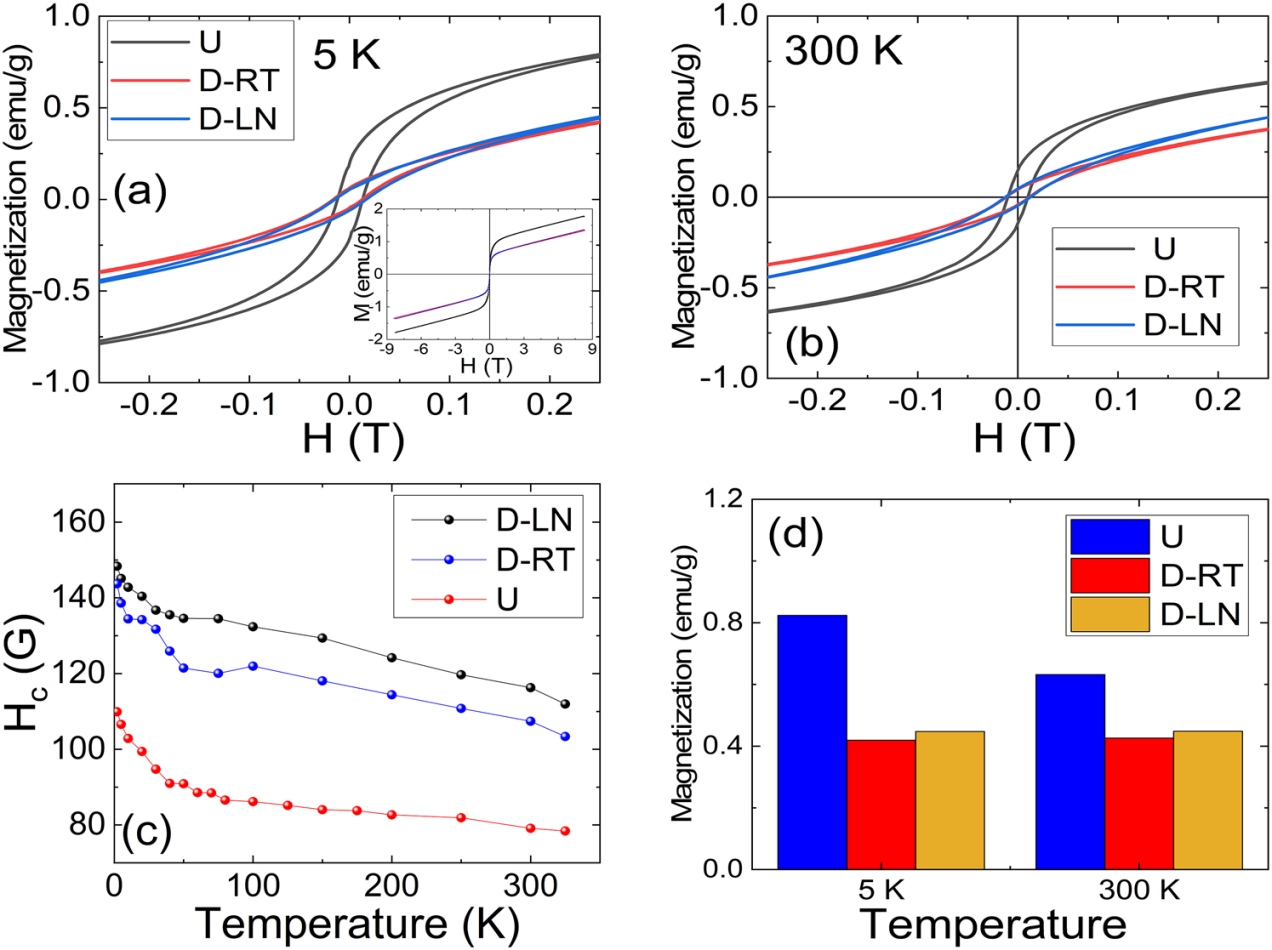
(**a**,**b**) Magnetic field dependence of the zero field cooled magnetization at indicated temperatures, Inset (**a**) high magnetic field measurements of the magnetic moments at the indicated temperature. (**c**) Temperature evolution of the coercive fields (**d**) Mass magnetization in a field of 0.25 T for undeformed and deformed samples.

One of the most important magnetic parameters for the applicability of this new group of materials is magnetic coercivity. The temperature evolutions of the coercive fields (H_C_) for undeformed and deformed samples have been determined as shown in Fig. 7. The evolution of H_C_ was extracted for all specimens as shown in Fig. 7c. As it can be seen from Fig. 7d, the magnetization significantly drops in deformed samples.

The undeformed specimen exhibited coercive fields between 108 and 78 gauss (8.6 kA/m and 6.2 kA/m) in the temperature range of 2 K and 350 K. Whereas the deformed samples exhibited significantly (around 60%) larger coercive fields for the same temperature range. Interestingly, the upturn observed at 70 K in the temperature dependence of the magnetization and its smearing out in deformed samples appear also in the coercive field data. Based on the extensive review of Gao et al*.*, the magnetic coercivity values reported for high entropy alloys fall into the category of soft to semi-hard magnetic materials^3,40,41^, and the values reported here fall into the category of semi hard-magnetic materials. It has been long realized that in magnetic alloys, the intrinsic defect structure, grain size and inclusions, and composition of the alloy have substantial effect on the coercivity of the material^42^. The coercivity increase can be attributed to the mechanical deformation induced residual stresses^43^. Similar behaviour has been the subject of many studies on Fe based steels where plastic deformation by tensile stress or cold-rolling engenders degradation of the soft magnetic behaviour of the material and subsequently leads to an increase of the coercive field and decrease of the permeability of the material. This has been attributed to the internal energy change due to the interaction between the local stress fields of domain walls and dislocations or other defects^44^. Note that the investigated samples in this work did not saturate even with fields as large as 9 T (see inset of Fig. 7a and supplementary data Fig. 1s) at all accessible temperatures. Field dependence of the magnetization grew almost linearly. Such behaviour has been observed by Krnel et al*.* and Kamarad et al*.* and interpreted as possible asperomagnetism or antiferromagnetism^14,45^.

In sum, the reported results revealed a very interesting magnetic structure: (1) A relatively abrupt increase in the magnetization was observed at 70 K for the undeformed sample while the change at this temperature was smeared out progressively for deformed samples. (2) Irreversibility in the temperature dependence of the ZFC and FC magnetization revealed evidence for magnetic frustration and clusters. (3) Field dependence of the magnetization indicated that the material acts differently than magnetically homogeneous ferromagnetic material where the magnetic moment is expected to saturate with an increasing magnetic field. In general, many magnetic states such as ferromagnetism^3,20,46^, ferrimagnetism^14,33^, antiferromagnetism^12^, and paramagnetism^47^ are present in high entropy alloys materials due to complex magnetic couplings of constituent *3d* elements. Often subtle compositional changes in 4 and 5 element systems stabilize one magnetic phase over the other. Hence, it is not completely surprising that magnetic phase coexistence and competition to be present in this group of relatively new systems. As discussed earlier, extremely low magnetic moments (0.0177 μ_B_/f.u. for the undeformed sample) suggest that the most possible ground state for the considered samples is the ferrimagnetic ground state. Similar to the current study, Kamarad et al. reported ferrimagnetic ground state for CrMnFeCoNi high entropy alloy with coexisting antiferromagnetic phase^14^. More specifically, in their work the linear increase of the magnetization with relatively large magnetic fields together with inverse susceptibility measurements were used to characterize this antiferromagnetic phase. In their case, the complex ferrimagnetic arrangement within the magnetic clusters is robust enough to withstand the large magnetic fields and subsequently do not reach saturation. Observation of non-saturating field dependence of magnetizations in this work at all accessible temperatures supports a similar conclusion for the considered FeMnCoCr alloy. Such behavior is not surprising as it is now well documented that Mn and Cr moments align antiferromagnetically with respect to Fe and Co ions^18^. The abrupt change in the temperature dependence of the magnetization for the undeformed sample (FCC) occurring at 70 K can be also interpreted within this frame: complex antiferromagnetic coupling between Mn, Cr, and Fe, Co weakens slightly with decreasing temperature and at around 70 K; a transition to a magnetically more ordered state takes place. Increased ferromagnetic coupling is evidenced also in the temperature dependence of the coercivity (see Fig. 7). Based on the recent DFT calculations by Niu et al*.,* the antiferromagnetic couplings are expected to be different in the HCP phase (enhanced), and hence the smearing out at 70 K in deformed samples is anticipated especially for the D-LN sample (HCP)^18^. Although the defect densities were not quantified in the current work, He et al*.* have reported similar levels of dislocation densities for Fe_40_Mn_40_Co_10_Cr_10_ at various deformation temperatures, including at 77 K^23^. This observation, along with the results presented in this work suggests that the magnetic ground state of this alloy system strongly depends on the defect density in addition to its structural phase.

**Figure 8:**
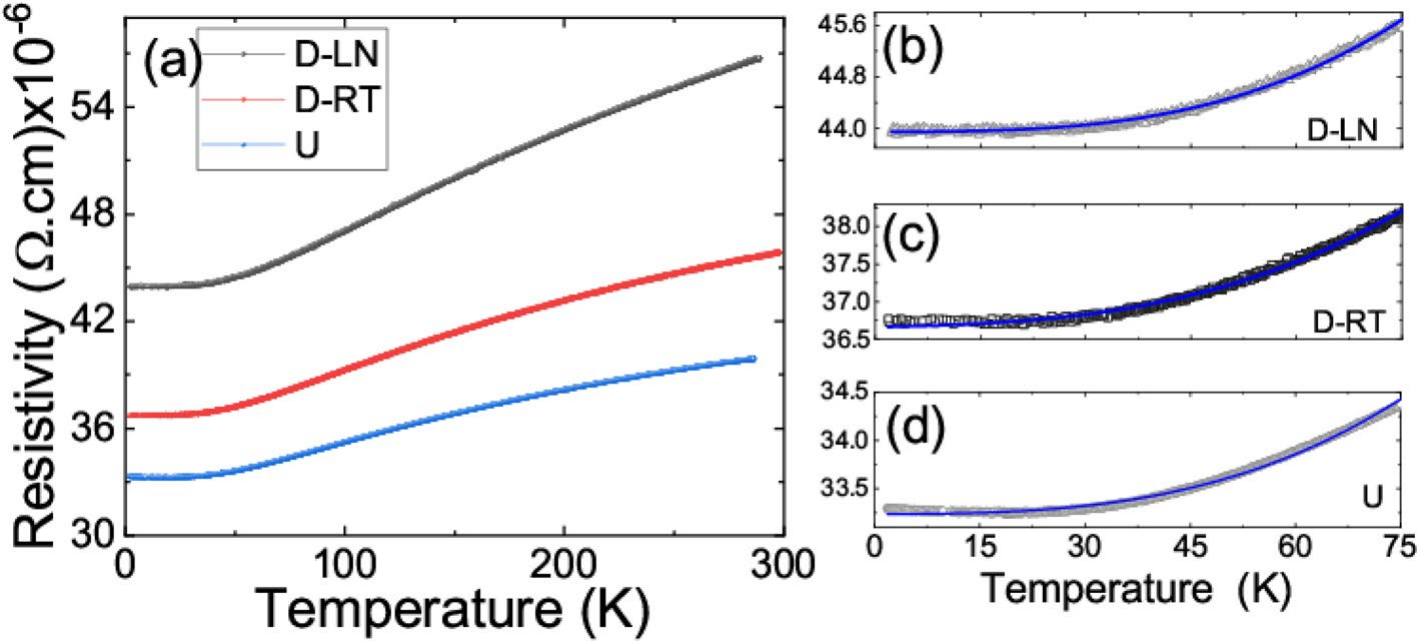
(**a**) Temperature dependence of the zero field resistivity of all samples. (**b**) Temperature dependence of the zero field resistivity for the temperature range of 2–75 K. Solid lines are fit to the power law.

In addition to the magnetization data, the temperature dependencies of the resistivity for all specimens were measured using the four point measurement method as shown in Fig. 8. The residual resistivity value increased significantly in deformed samples. In particular, roughly a 10% and 25% increase of the residual resistivity has been observed for RT and LN deformed samples, respectively. However, the overall shape and trend of the curves remained the same which is indicative of a similar conduction mechanism. The increase in resistivity is rather expected as deformed specimens have larger defect densities compared to the undeformed sample^48^. Interestingly, the temperature dependence of the resistivity measurements reveals a residual resistivity ratio (RRR = R(300 K)/R(2 K)) of 1.15 for the undeformed sample and 1.25 and 1.30 for the RT and LN deformed specimens, respectively. These values are very typical for similar materials in the general context of high entropy alloys^16^. The temperature dependence of the resistance has been fitted to power law models for various high entropy alloys^16,17^. R (T) = R_0_ + R_2_T^2^ + R_3_T^3^ + R_F_T^0.5^ has been used for fittings. In this expression R_0_, R_2_, R_3_, and R_F_ are resistive constants and T is the temperature. In particular, R_0_ represents the residual resistivity of the studied material which is generally linked to the imperfections and impurities in the sample. Note that resistive constants obtained for the undeformed sample will represent the case for the pure FCC phase while the resistive constants determined for deformed samples will represent the average for FCC and HCP phases. A T^2^ dependence of the R(T) is a typical feature in metallic systems and is often interpreted to arise from electron–electron scattering events. Cubic (T^3^) dependence of the resistance on temperature is traditionally considered as indicative of electron–phonon scattering events as the main mechanism in the description of resistance. Square root (T^0.5^) temperature dependence has been used to account for temperature dependent disorder induced electron–electron scattering events^17^. Square root temperature dependence of resistivity, which when combined with the normal T^2^ or T^3^ contribution results in a resistivity minimum, well fits the reported data for the low temperature range (see Fig. 8). All the resistive constants in the fits can be found in the supplementary data document. Note that Kondo like scattering term, ln(1/T) dependence has been also introduced to account for the low temperature upturn in the temperature dependence of the resistivity similar to ones observed here^17^. However, we believe, relating subtle upturn in the temperature dependence of the resistivity to intrinsic defect structure of the material is more likely than the Kondo scattering since we have observed bulk magnetism in our samples. It is worth mentioning here that no significant magnetoresistance was observed (up to magnetic fields of 9 T) in any of the samples at any temperature within the measurement range. In magnetic materials, one will expect to observe magnetoresistive effects due to anisotropic magnetoresistance and magnetic impurity scattering. Indeed, to the author’s knowledge, 3d transition metal based high entropy alloys exhibit very small magnetoresistance with a couple of exception like the AlCrFeCoNiCu system^15,49^. Moreover, there are number of reports showing significant magnetoresistance in rare earth elements based on high entropy alloys^45,50^.

## Summary

In summary, a prototypical high entropy alloy Fe_40_Mn_40_Co_10_Cr_10_ has been mechanically deformed at room temperature and liquid nitrogen temperatures. A significant enhancement in strength was observed at cryogenic deformation temperatures. For example, the yield strength increased from around 150 MPa at RT to 340 MPa at LN (≈130% increase). The deformation was accommodated by crystallographic slip at RT and primarily through phase transformation (TRIP) at LN. A detailed electron back scatter diffraction analysis allowed us to obtain critical information on the onset and evolution of the HCP phase as a function of strain. The incremental accumulation of the HCP phase, and therefore the higher phase-boundary density, and the associated increase in dislocation density, improves the hardening capacity thus allowing for high ductility levels. We have elucidated the effect of deformation temperature, which consequently alters the deformation mechanism from slip dominated at 298 K to TRIP dominated at 77 K, on the physical properties of this alloy system. The measured magnetic moment for the undeformed sample at 2 K was around 1.8 emu/g in a field of 9 T while it is only 0.4 emu/g in a field of 0.05 T. The magnetization dropped significantly for the deformed specimens. In the temperature dependence of the magnetization, there is a clear upturn on setting around 70 K and reaching to saturation at around 40 K. Temperature dependence of the magnetization is almost flat between 70 and 290 K with a slight downward turn in magnetization around 290 K. The clear abrupt transition observed in the undeformed state at 70 K was smeared out progressively for D-LN and D-RT samples and interpreted as an enhancement of the antiferromagnetic interactions in the deformed samples. There is a peak in temperature dependence of the ZFC magnetizations in the low field regime indicative of possible magnetic frustration. Note that the deformation will result in elongation in grain size in the loading direction. Although EBSD images from the deformed region do not indicate a large variation in grain size, such an effect can alter the magnetic properties significantly. Hence, the enhanced irreversibility observed in deformed samples can be also interpreted as the change in the magneto crystalline anisotropy of the specimens. The magnitudes of the coercive fields places the considered FeMnCoCr material in to semi-hard magnetic alloys category. The coercivity of the deformed samples are significantly higher than the undeformed sample. Temperature dependence of the resistivity revealed residual resistivity values of 34 μΩ.cm for the undeformed sample and 38 μΩ.cm and 44 μΩ.cm for RT and LN deformed samples respectively. Moreover, the residual resistivity ratio increased in deformed samples. Considering the fact that the undeformed sample has only the FCC phase and LN deformed samples have both FCC and HCP phases based on the EBSD data, one can conclude that the HCP phase is more resistive than the FCC phase. The relatively small magnetic moments along with non-saturating magnetic fields suggest that the ground state in the considered specimens is ferrimagnetic ordering with coexisting antiferromagnetic phase. The gradual smearing out of the abrupt transition at 70 K (measured in low magnetic fields), is potentially induced by an increased antiferromagnetic coupling in deformed samples especially in the HCP sample. Due to the limitations in the utilized experimental setup, the magnetic ordering temperatures for the undeformed and deformed samples were not established in the current work. However, the data presented in Fig. 5 suggest that the magnetic ordering temperature of the undeformed and deformed samples could be significantly different. The presented data supports the conclusion that the structural phase is not the only important aspect in defining the magnetic and electrical properties of this group of materials but also the defect density must be considered.

## Methods

Fe_40_Mn_40_Co_10_Cr_10_ (at.%) alloy ingot was arc-melted using pure elements (purity > 99.5 wt.%). Following casting, the ingot was homogenized at 1200 °C for 24 h in an inert environment. All specimens investigated in this work were machined from the homogenized ingot using electric wire EDM. Specimens were solution heat treated at 900 °C for 1 h followed by quenching in oil to assure a single phase, face-centered cubic (FCC) structure. EDM surface damage and any oxidation were removed during sample preparation as detailed in Ref.^51^. The crystal structure, phase purity and chemical homogeneity of the studied material were analysed by Panalytical X’pert^3^ powder X-ray diffraction (XRD) and further complemented by energy dispersive X-ray spectroscopy (EDX) and electron backscatter diffraction (EBSD) measurements. EBSD and EDX measurements were collected using a JOEL 7000F equipped with HKL EBSD detector. For mechanical properties characterization, dog bone specimens (3 × 1.5 mm^2^ cross section and 8 mm gauge length) were deformed in tension at two deformation temperatures, 298 K (i.e., room temperature RT) and 77 K (LN temperature). For the experiments at 77 K, both the specimen and the grips were submerged in liquid nitrogen throughout the experiment. Incremental deformation was applied using an Instron load frame, in displacement control, with an average strain rate of ≈ 4 × 10^–1^ s^−1^. High resolution strain measurements and EBSD were collected following unloading at different levels of deformation. For each of the considered deformation temperatures, specimens were eventually loaded to about the same level of total plastic strain, 22.1% and 22.5% for the RT and LN specimens, respectively. Physical properties were collected at this level of deformation for both the RT and LN deformed samples as well as undeformed conditions. We have used bar shaped specimens of 2.5 × 1.0 × 1.0 mm^3^ for magnetization measurements. For the deformed specimens, the bar was cut from the central area similar to one shown in Fig. 3b (red area). Field were always applied along one of the short axis which corresponds to the loading direction. Here we have to mention that our specimens (as revealed from the high resolution EBSD images) consists of very large grains (200–250 μm) hence directional dependence of the magnetic properties are present in our specimens. We have put extra effort to start with exactly identical sample with (same magnetic moment and temperature dependence) to avoid this intrinsic anisotropy effects.

Magnetic and electrical transport properties were measured using a Cryogenics ltd. high field measurement system with vibrating sample magnetometer option in a cryogenic physical property measurement system. Cryostat was closed cycle helium cryostat with variable temperature insert (VTI) in which the VTI and samples temperatures measured and controlled with a tolerance of 5 mK. The Electric transport properties were measured using 4 probe method which uses a Keithley 2182A nanovoltmeter and Keithley 2461 source measure unit in delta mode for highest sensitivity. Mechanical tests were performed at room temperature (RT) and liquid Nitrogen (LN) temperatures. Our discussion will concentrate on undeformed sample as well as deformed samples. Hence for convenience in this manuscript we refer these samples as U for undeformed sample, D-RT for room temperature deformed sample and D-LN for liquid nitrogen deformed sample.

## Supplementary Information

Supplementary Information.
