# Supplementary material for: Magnetic, electrical and mechanical properties of Fe40Mn40Co10Cr10 high entropy alloy
Source: Sci Rep. 2021 Apr 13;11:8048. doi: 10.1038/s41598-021-87527-x (PMC8044135; doi:10.1038/s41598-021-87527-x)
Supplement: Supplementary file 1 — Supplementary Information. [file 41598_2021_87527_MOESM1_ESM.docx]

Supplementary Data for:

**Magnetic, Electrical and Mechanical properties of Fe_40_Mn_40_Co_10_Cr_10_ Medium Entropy Alloy**

M. Egilmez^1,*^, W. Abuzaid^2, #^

*1. Department of Physics, American University of Sharjah, UAE.*

*2. Department of Mechanical Engineering, American University of Sharjah, UAE.*

*** [*megilmez@aus.edu*](mailto:megilmez@aus.edu)

*^#^* [*wabuzaid@aus.edu*](mailto:wabuzaid@aus.edu)

**1-Resistive Constants**

Table S1. As mentioned in the text the resistivity data were fit to R (T) = R_0_+ R_2_T^2^+ R_3_T^3^+R_F_T^0.5^ expression. Fitting constants are below.


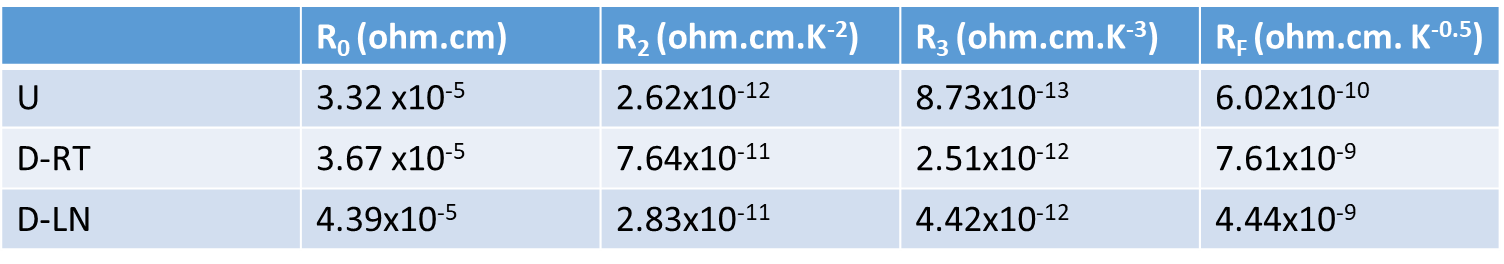


**2-Field dependence of the magnetization:**

Figure 1s represents the magnetic field dependence of the magnetization data for undeformed and deformed samples. At all temperatures, all the samples exhibit magnetization trends that grow linearly with increasing magnetic fields.


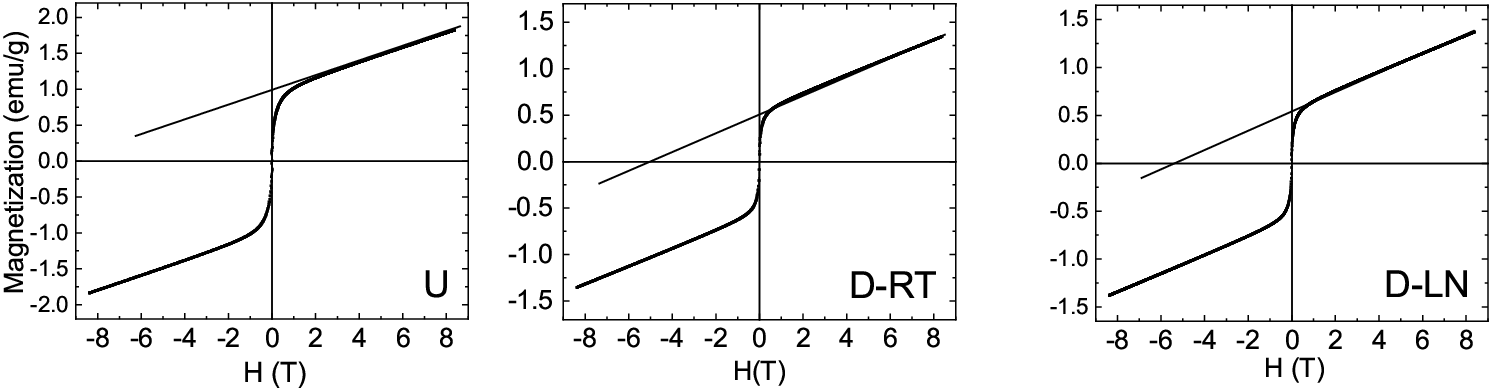


F**igure 1s:** High field range hysteresis loops for all samples at 325 K. Lines are guide for eye.

**3. Temperature dependence of the inverse magnetization.**


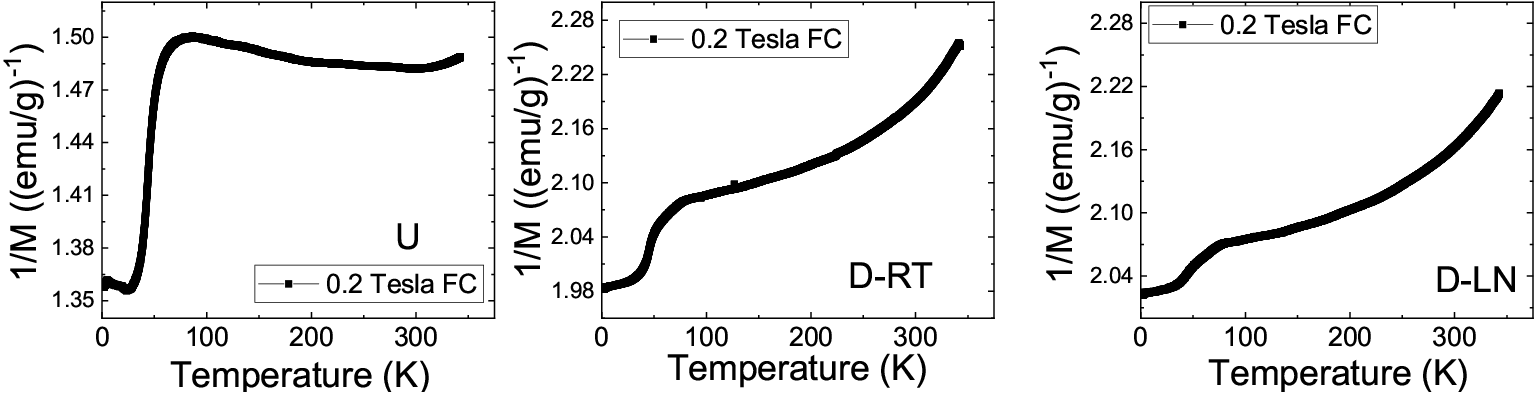


F**igure 2s:** Temperature dependence of the inverse magnetization for deformed and undeformed samples.
